# Hypoglycemic Effect of *Laminaria japonica* Polysaccharide in a Type 2 Diabetes Mellitus Mouse Model

**DOI:** 10.5402/2012/507462

**Published:** 2012-10-30

**Authors:** Xiaodan Li, Zhuqin Yu, Shaohua Long, Yunliang Guo, Delin Duan

**Affiliations:** ^1^Affiliated Hospital, Medical College Qingdao University, Shandong, Qingdao 266003, China; ^2^Institute of Oceanology, Chinese Academy of Sciences, Shandong, Qingdao 266071, China

## Abstract

The aim is to investigate the hypoglycemic effect of *Laminaria japonica* polysaccharides (LJPS) on type 2 diabetes mellitus (T2DM) mice model. 60 healthy male mice have been used in the experiment. T2DM animal mode was prepared by high fatty forage feeding and intraperitoneal injection with alloxan. Diabetic mice were orally supplied with LJPS. Then their blood was collected for various biomedical measurements of fasting blood glucose (FBG), serum insulin, and amylin. Treatment with LJPS significantly reduced fasting blood glucose (*P* < 0.05) and increased the levels of insulin and amylin in serum (*P* < 0.05). Overall, the study presented that LJPS can reverse several components of T2DM. Therefore, LJPS may become a new oral candidate medicine for the treatment of diabetes.

## 1. Introduction

Type 2 diabetes mellitus (T2DM) is a chronic, progressive metabolic disease that involves multiple factors such as age, life style, genetic factor, obesity and viral infection [1–5]. The pathogenesis of T2DM includes insulin resistance (IR), glucotoxicity, lipotoxicity, oxidative stress, genetic deficiency and inflammatory reaction among which, IR is crucially important occupying the central position in this metabolic syndrome [6–9]. The “common soil hypothesis” theory [10] considers IR a common pathogenic factor for coronary heart disease, diabetes, and hypertension. Amylin secreted from the pancreatic islets into the blood circulation contributes to glucose metabolism control in physiological condition [11]. But, long-term hyperglycemia stimulated to induce the production of amylin that can inhibit insulin releasing, also inhibit insulin-stimulated glucose transport in skeletal muscle and the glucose metabolism of liver cell. The turbulence of excretion of amylin induces insulin resistance, interfering with fat metabolism [12]. The current therapies of type 2 DM include increasing secretion of islet cells, improving insulin sensitivity to peripheral tissue, reducing glucose absorption in the gastrointestinal tract and decreasing serum lipid and increasing insulin level. However, drug susceptibility declines with long-term use and toxic side effects are accompanied by increased dosage. Therefore, new and much more effective medical therapies must be developed to improve the treatment and protection of the patients with T2DM. Inhibitors of glucagon and amylin analogues may avoid the above shortcomings to some degree [13].

A traditional Chinese medicine, named Kelp, was used in the present study as an anti-T2DM drug. The main effective components of this kelp are *Laminaria japonica* polysaccharides (LJPS), including *Alginate*, *Fucoidan,* and *Laminaran* [14]. It has been reported that LJPS has a variety of effects, such as antioxidant and free radicals scavenging effects, antitumor efficacy, reducing blood lipids function, improving immunity and antithrombotic effects [15–18]. However, only scarce research aimed to study the hypoglycemic effect of LJPS on diabetes [19]. For this study, we investigated the effect of LJPS on type 2 DM mouse model that was induced by fat-rich forage and intraperitoneal injection with alloxan.

## 2. Materials and Methods

### 2.1. Mouse Model

 All experimental procedures were in accordance with guidelines for the care and use of laboratory animals were approved by the Ethics Committee of Qingdao University Medical College (no. QUMC 2011-09). Sixty healthy male Kunming mice weighing 23–27 g were purchased from the Experiment Animal Center of Qingdao Drug Inspection Institute (SCXK (LU) 20110010). Animals were acclimatized to feed with normal forage for 7 days. Ten mice were randomly selected as a control group and given general forage. The remaining 50 mice were fed with high fatty forage composed of general forage (59%), sucrose (20%), pig fat oil (10%), egg yolk powder (10%), and cholic acid sodium (1%) [20]. After 4 weeks of dietary manipulation, alloxan (50 mg/kg body weight) was given by intraperitoneal injection once every other day for 3 times to establish type 2 MD models [21]. Mice in the control group were administered with equivalent amounts of normal saline. Fasting blood glucose (FBG) was measured third day after the final injection. The type 2 DM animal model as the successful markers for establishing model was when FBG differed by more than two standard deviations from the control group. Ten experimental mice were excluded because they did not satisfy the standard. The remaining 40 type 2 DM model mice were divided randomly into model group (*n* = 10) and treatment groups including a low-dose group (*n* = 10), a medium-dose group (*n* = 10) and a high-dose group (*n* = 10).

### 2.2. Preparation of LJPS

LJPS was extracted from “Zhongke No.1” *Laminaria japonica* harvested in Rongcheng, Shandong, China. The *Laminaria japonica* was pulverized to powder, and by using the ultrasonic extractor, filtered and concentrated. Protein was removed by the Sevage method. The extract was washed repeatedly with ethanol and acetone, and then frozen and dried. The yield of fine LJPS accounted for 17.9% (w/w) compared with the original *Laminaria japonica*.

LJPS was diluted with normal saline to desired concentrations (75 mg/mL [low dose], 150 mg/mL [medium dose], 300 mg/mL [high dose]). Diabetic Mice were treated by orally administration respectively using 75 mg/mL [low dose 0.75 g/kg], 150 mg/mL [medium dose 1.50 g/kg], 300 mg/mL [high dose 3.00 g/kg] LJPS once a day for 2 weeks. In the control group and model group Mice were orally administrated an equivalent amount of saline. Meanwhile, all of animal model mice were fed normal forage for 2 weeks.

### 2.3. Blood Sampling and Analysis

Blood samples from each mouse were collected and centrifuged for 10 minutes at 4000 r/min to separate serum which was then stored at −20°C until use.

FBG was measured with an automatic blood glucose meter (Roche Diagnostics, Shanghai Co., Ltd.) and blood glucose test strips (ACCU-CHEK Perform test strips). Insulin was analyzed using Elecsys 2010 and Cobase 411 analyzers and Roche diagnostics reagent kits (12017547). All experimental assays were done according to the manufacture's manual. Assay measurement range was 1.39–6945 pmol/L. Enzyme linked immunosorbent assay (ELISA) (Andy Sci. & Tech. Co., Ltd., USA) determined amylin serum level. Assay sensitivity was 0.01 ng/mL for amylin serum level.

### 2.4. Statistical Analysis

SPSS17.0 software was used for statistical analysis and data were expressed as $\overset{-}{x}\pm S$. Multi-group comparison was via analysis of variance (ANOVA) and Student's test and two-group comparison was by *t*-test. Values were considered to be significant when *P* < 0.05.

## 3. Results

### 3.1. Fasting Blood Glucose (FBG)

FBG of rats had no remarkable difference comparing with the controls before the experiment, (*P* > 0.05). But, FBG had obvious difference by analysis of varance in every group before treatment (*F* = 14.32, *q* = 0.01 ~ 1.57, *P* < 0.05), and model group had bilateral statistical difference compared with controls (*t* = 2.64, *P* < 0.05). After four weeks of LJPS treatment was significantly decrease FBG and unilateral statistics compared with molds (*F* = 4.02, *q* = 0.01 ~ 2.94, *P* < 0.05) (*t* = 1.896, *P* < 0.05), though there was no significant difference among treatment groups (*P* > 0.05) as showed in Table 1.

### 3.2. Serum Insulin and Amylin

There was significant difference of insulin serum level among model group and treatment groups (*F* = 15.62, *q* = 4.12–12.73, *P* < 0.05). Serum insulin was significantly increased by LJPS oral administration (*t* = 5.53, *P* < 0.05). The serum amylin differed significantly among all groups (*F* = 13.96, *q* = 0.00–12.38, *P* < 0.05); the serum amylin of the low dose group was higher than that in the model group (*t* = 5.53, *P* < 0.05).  Table 2 showed that there was no significant difference among treatment groups (*P* > 0.05).

## 4. Discussion

Traditional Chinese medicine has been reported for a long time to have antidiabetic efficacy and fewer side effects compared to existing drugs. Therefore, treatment of diabetes with Chinese traditional medicine has been increasing rapidly [22–24]. The present study has demonstrated the effect of Chinese traditional medicine LJPS on T2DM.

The high-fatty forage and intraperitoneal injections with alloxan induced type 2 diabetic mouse model mimicks the natural history of the metabolic features of human type 2 diabetes [19, 20]. For this study, the model group exhibited significant increase of serum glucose compared with normal control group. In addition, insulin and amylin in serum levels were reduced after alloxan injection and long periods of high fat diet. These results suggested the successful establishment of type 2 diabetes mouse model. 

Treatment with 8 weeks of LJPS reduced FBG levels compared with model group. This indicates that LJPS has hypoglycemic effect in type 2 DM mice model. In general, low dosage of LJPS exhibited the best results. Furthermore, LJPS treatments also increased insulin and amylin compared with model group. Metabolic disorder could appear diabetes mellitus symptom when a body has high levels of glucose, fat, and cholesterol in the long-term [5]. After an long period of consuming food with high sugar, high fat, and high cholesterol and alloxan of injection, mice suffered from type 2 DM. Alloxan has specific toxic effect on beta cells of pancreatic island. Such toxic effect could be due to production of oxygen free radicals in pancreas which damage DNA and mRNA in islets, leading to reduced synthesis of insulin and amylin of beta cells [25]. Previous studies reported that LJPS could reduce serum lipid in hyperglycemia rats through its antioxidation properties [26] and could have antioxidant and hypoglycemic effects on diabetes mellitus induced by alloxan in rats [21] which the present study is consistent with. In this study, increased insulin an amylin indicates that LJPS could partially recover the secretory function of islet cells by antioxidant effects. Further studies will be required to investigate the direct effect of LJPS on islet cells.

The present experiment demonstrated that insulin and amylin levels in serum of model group mice decreased comparison with controls, indicating that islet beta cells may be damaged in early stage of type 2 DM. After LJPS treatment, FBG was decreased significantly, serum insulin and amylin levels were elevated in diabetes mellitus mouse suggests that LJPS has hypoglycemic effect in type 2 DM and that LJPS may partially recover the secretary function of islet cells, leading to elevated serum levels of insulin and amylin and improved glucose metabolism regulation. This study showed no significant difference in FBG, serum insulin and amylin levels among treatment groups, suggesting that the hypoglycemic effect for treating type 2 DM could be achieved with low-dose LJPS (0.75 g/kg). Further studies needs to be carried out to elucidate precise mechanisms of this effect. 

## 5. Conclusions

In summary, the present study has reported the beneficial impact of LJPS on T2DM in mice. The hypoglycemic effects by LJPS may due to regulation of insulin and amylin in serum.

## Figures and Tables

**Table 1:** Effects of LJPS on FBG ($\bar{x}\pm S$, mmol/L).

Groups	*n *	Dose	Original FBG	FBG before LJPS treatment	FBG after LJPS treatment
Control group	10	NS	7.40 ± 1.23	6.70 ± 1.05	6.19 ± 1.27
Model group	10	NS	7.40 ± 1.23	10.18 ± 0.97^ab^	8.47 ± 0.91^b^
Low dose group	10	0.75 g/kg	7.40 ± 1.23	9.90 ± 1.01^a^	7.34 ± 1.17^c^
Medium dose group	10	1.50 g/kg	7.40 ± 1.23	9.80 ± 1.48^a^	6.08 ± 1.27^c^
High dose group	10	3.00 g/kg	7.40 ± 1.23	9.95 ± 1.03^a^	6.86 ± 1.46^c^

^a^ *P* < 0.05 versus before modeling, ^b^ *P* < 0.05 versus control group; ^c^ *P* < 0.05 versus model group.

**Table 2:** Animal index levels after LJPS treatment ($\bar{x}\pm S$).

Groups	*N *	Dose	Insulin (pmol/L)	Amylin (ng/L)
Control group	10	NS	79.23 ± 13.62	19.34 ± 0.57
Model group	10	NS	15.73 ± 4.64^a^	15.43 ± 1.85^a^
Low dose group	10	0.75 g/kg	28.16 ± 5.38^b^	23.08 ± 2.54^b^
Medium dose group	10	1.50 g/kg	27.83 ± 5.16^b^	22.21 ± 1.78^b^
High dose group	10	3.00 g/kg	31.14 ± 6.12^b^	24.90 ± 4.01^b^

^a^ *P* < 0.05 versus control group, ^b^ *P* < 0.05 versus model group.
